# Prion Protein Amino Acid Determinants of Differential Susceptibility and Molecular Feature of Prion Strains in Mice and Voles

**DOI:** 10.1371/journal.ppat.1000113

**Published:** 2008-07-25

**Authors:** Umberto Agrimi, Romolo Nonno, Giacomo Dell'Omo, Michele Angelo Di Bari, Michela Conte, Barbara Chiappini, Elena Esposito, Giovanni Di Guardo, Otto Windl, Gabriele Vaccari, Hans-Peter Lipp

**Affiliations:** 1 Department of Veterinary Public Health and Food Safety, Istituto Superiore di Sanità, Rome, Italy; 2 Institute of Anatomy and Center for Neuroscience, University of Zürich, Zürich, Switzerland; 3 Department of Comparative Biomedical Sciences, Faculty of Veterinary Medicine, University of Teramo, Teramo, Italy; 4 Veterinary Laboratory Agency, TSE Molecular Biology Department, Weybridge, New Haw, Addlestone, Surrey, United Kingdom; University of Alberta, Canada

## Abstract

The bank vole is a rodent susceptible to different prion strains from humans and various animal species. We analyzed the transmission features of different prions in a panel of seven rodent species which showed various degrees of phylogenetic affinity and specific prion protein (PrP) sequence divergences in order to investigate the basis of vole susceptibility in comparison to other rodent models. At first, we found a differential susceptibility of bank and field voles compared to C57Bl/6 and wood mice. Voles showed high susceptibility to sheep scrapie but were resistant to bovine spongiform encephalopathy, whereas C57Bl/6 and wood mice displayed opposite features. Infection with mouse-adapted scrapie 139A was faster in voles than in C57Bl/6 and wood mice. Moreover, a glycoprofile change was observed in voles, which was reverted upon back passage to mice. All strains replicated much faster in voles than in mice after adapting to the new species. PrP sequence comparison indicated a correlation between the transmission patterns and amino acids at positions 154 and 169 (Y and S in mice, N and N in voles). This correlation was confirmed when inoculating three additional rodent species: gerbils, spiny mice and oldfield mice with sheep scrapie and 139A. These rodents were chosen because oldfield mice do have the 154N and 169N substitutions, whereas gerbil and spiny mice do not have them. Our results suggest that PrP residues 154 and 169 drive the susceptibility, molecular phenotype and replication rate of prion strains in rodents. This might have implications for the assessment of host range and molecular traceability of prion strains, as well as for the development of improved animal models for prion diseases.

## Author Summary

Prions are unconventional infectious agents that cause fatal neurodegenerative diseases in animals and humans. A pathological form of the cellular prion protein (PrP^C^), named PrP^Sc^, appears to be the major or the sole component of prions. These agents are transmitted by inducing the conversion of host PrP^C^ into PrP^Sc^ that accumulates in the brain of affected individuals. Different factors are believed to modulate such events, which explains the variable transmission efficiency observed under inter-species experimental inoculation. These factors are still fairly unknown, although evidence exists that some kind of structural compatibility between PrP^Sc^ of the infectious inoculum and PrP^C^ of the host has a role in making transmission more or less efficient. We investigated the transmission of prions to different rodents and showed that specific amino acid substitutions (Y154N and S169N) in the prion protein are major determinants of susceptibility to prions. In particular, we showed that these specific variations i) direct the transmission rate of prions between different species in a way that is dependent on the prion strain, ii) affect the molecular characteristics of prions, and iii) influence their replication efficiency.

## Introduction

The conversion of the cellular prion protein (PrP^C^) into an abnormally-folded isoform (PrP^Sc^) that accumulates in the brain of affected individuals represents the key feature of transmissible spongiform encephalopathies (TSEs), or prion diseases [1]. They include bovine spongiform encephalopathy (BSE) in cattle, scrapie in sheep, Creutzfeldt-Jakob disease (CJD) and variant CJD (vCJD) in humans. According to the “prion theory”, PrP^Sc^ is the major or the sole component of the TSE agents, named prions. These unusual agents are believed to self-propagate by catalyzing the conversion of PrP^C^ into PrP^Sc^ which acts as a template [2].

Experimental animals are of paramount importance for the study of TSEs. However, very long incubation periods or even unsuccessful transmissions are observed when a given model is challenged with prions from a different species. Prion transmission to a new species is in fact limited by a phenomenon known as “species barrier” [3].

Early studies argued that the main factor influencing interspecies transmission resides in the homology degree of the amino acid sequence of PrP between the donor and recipient species [4]. Differences in the PrP sequence can result in a non-effective interaction between PrP^Sc^ and PrP^C^ and in an inefficient propagation of PrP^Sc^ that produce prolonged incubation periods. The sequence of the donor PrP^Sc^ and host PrP^C^ is identical on second passage in the same species and this can explain the adaptation of the agent to the new host, which results in shorter incubation periods. Transgenic mice carrying the PrP gene of the donor species have been generated with the aim of removing the transmission barrier. These models provided evidence that not only PrP homology but also the prion strain played a prominent role in the transmission barrier. Although highly susceptible to sporadic CJD, transgenic mice over-expressing human PrP showed lower susceptibility to vCJD than wild-type mice [5]. Individual strains represent different PrP^Sc^ conformations within the framework of the “prion theory” [6].

Recently, we reported that the interspecies transmission of prions from humans to bank voles (*Myodes glareolus*) can occur without an apparent species barrier despite a low degree of PrP sequence homology between voles and humans [7].

Studies of transmission barrier are important for elucidating the basis of prions replication and acquiring knowledge to decipher the risk of interspecies transmission. The availability of animal models susceptible to different prion strains is of crucial relevance for such kind of studies.

We recently showed that the bank vole is very susceptible to TSEs [7],[8],[9]. Here, we studied the transmission features of different TSEs in a panel of seven rodent species showing various degrees of phylogenetic affinity and specific PrP sequence divergences in order to investigate the molecular basis of the high susceptibility of voles in inter- and intra-specific transmissions.

## Results

### Inverted susceptibility of mice and voles to natural scrapie and BSE

Transmission studies were first set up in bank and field voles in comparison to C57Bl/6 and wood mice. Concerning natural scrapie (SS3), the results of primary transmission to bank voles and C57Bl/6 mice were previously reported [9].

One hundred per cent of bank and field voles developed obvious clinical signs and were sacrificed after short survival times following inoculation of natural scrapie (Table 1). The first signs of disease in both vole species were hyperactivity/reactivity followed by the progressive disappearance of the typical behaviour of hiding under the cage's sawdust. Overt neurological signs appeared later and consisted of incessant walking along the cage and characteristic upward movements of the head (head bobbing), accompanied by severe and progressive ataxia. Hunched posture, apathy and pronounced hypo-activity/reactivity preceded sacrifice or death which occurred 10–20 days after the onset of neurological signs.

**Table 1:** Primary transmission and second passage of natural sheep scrapie (SS3) and BSE to bank voles, field voles, C57Bl/6 mice and wood mice.

Recipient species	Inoculum	Primary transmission					Second passage	
		Clin. signs (+)	Pathol. (+)	PrP^Sc^ (+)	Survival time (days±SD)	Transm. rate (%)	PrP^Sc^ (+)/inoculated	Survival time (days±SD)
Bank voles	SS3§	9/9	9/9	9/9	199±28	100	7/7	92±14
	BSE	0/6	0/4	0/6	>1044*	0	6/6	483±85
Field voles	SS3	8/8	8/8	8/8	259±53	100	n.d.	n.d.
	BSE	0/10	0/8	0/5	>707*	0	n.d.	n.d.
Wood mice	SS3	0/7	0/7	0/7	>1341*	0	n.d	n.d
	BSE	9/9	8/8	9/9	720±38	100	n.d.	n.d.
C57Bl/6 mice	SS3§	0/9	3/9	3/9	567±149°	33°	16/16	233±9
	BSE	4/7	6/7	6/7	631±25°	86°	10/10	194±2

**§:** Results of the transmission of SS3 to bank voles and C57Bl/6 mice were previously reported [9].

*: When no PrP^Sc^-positive animal was found in the group, survival time is shown as longer (>) than the survival time of the last sacrificed/dead animal.

**°:** Survival times and transmission rates were calculated only on animals showing PrP^Sc^ accumulation.

In contrast, the inoculation of natural scrapie in C57Bl/6 and wood mice produced very long survival times without overtly suggestive signs of prion disease. C57Bl/6 mice rarely showed subtle and equivocal signs such as nervousness and hyper- reactivity, followed by apathy.

PrP^Sc^ brain accumulation was detected by Western blot in 100% of bank and field voles whereas it was not detected in wood mice and in only three out of nine C57Bl/6 mice (Table 1).

The reverse situation was apparent following the inoculation of BSE. Neither of the two vole species showed clinical signs or PrP^Sc^ accumulation during their lifetime. This is in contrast to C57Bl/6 and wood mice which showed overt neurological signs characterized by hyper-activity/reactivity and followed by hind limb incoordination, hunched posture and apathy. Survival times were rather long in both mice species, but the transmission rate was high (Table 1).

**Figure 1:**
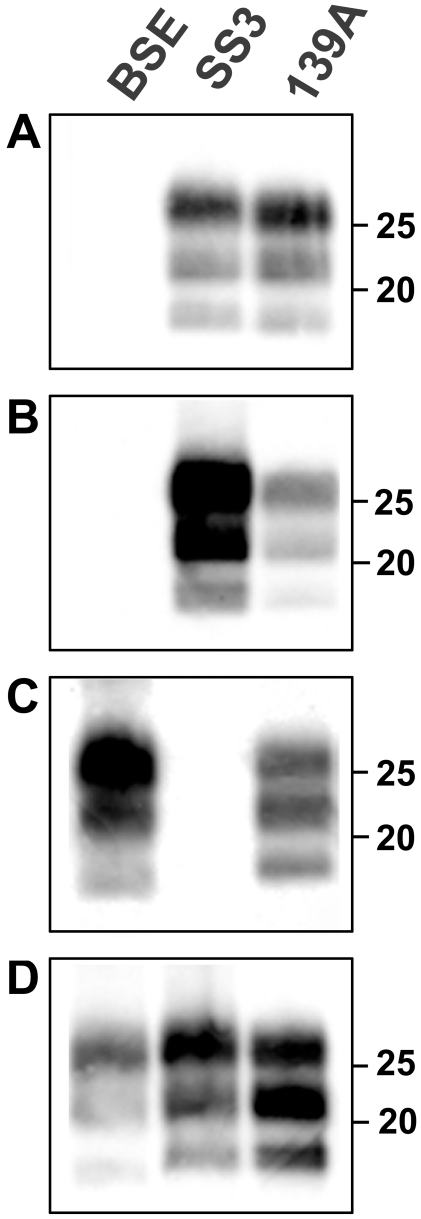
Immunoblot of PrP^Sc^ from the primary transmission of prion strains to rodent species. (A) bank voles, (B) field voles, (C) wood mice and (D) C57Bl/6 mice.

**Figure 2:**
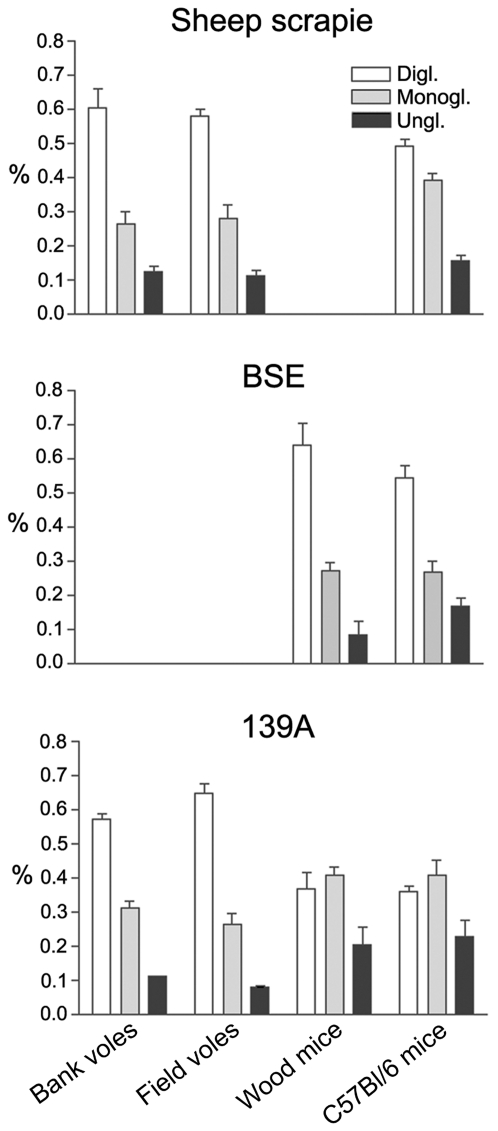
Glycoform analysis of PrP^Sc^ from rodent species inoculated with SS3, BSE and 139A.

Western blot showed that the apparent molecular weight (MW) of proteinase K-treated PrP^Sc^ from BSE and natural scrapie was maintained after transmission to rodents. Indeed, the unglycosylated isoform of PrP^Sc^ from BSE was ∼1 kDa lower than scrapie (Fig. 1). Glycoform analysis showed the typical three bands whose rank order of density was diglycosylated>monoglycosylated>unglycosylated in all species and with both TSE sources (Fig. 2). The BSE glycoprofile in C57Bl/6 and wood mice was characterized by a very high intensity of the diglycosylated isoform.

**Figure 3:**
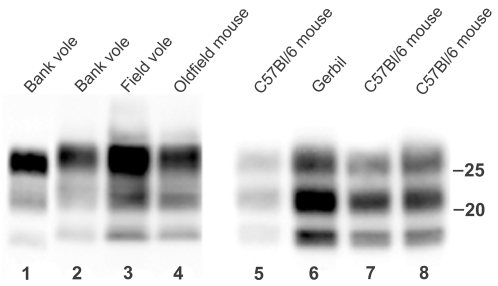
Molecular type of PrP^Sc^ produced in rodent species following transmission of prion strains. Immunoblot of PrP^Sc^ from the second passage of BSE to bank voles (lane 1), the primary transmission of 139A to bank voles, field voles, oldfield mice and gerbils (lanes 2, 3, 4, 6), the transmission of 139A to C57Bl/6 mice (lanes 7, 8) and the back passage of vole-adapted 139A to C57Bl/6 mice (lane 5).

Second passages were performed on C57Bl/6 mice and bank voles, which were considered representative of the two rodent groups. They were successful in all inoculated animals (Table 1). Survival times shortened compared to primary transmissions, which demonstrated the existence of obvious transmission barriers. Despite the lack of any evidence of BSE transmission to voles upon primary passage, a second “blind” passage of BSE was carried out by using the brain of the individual showing the longest survival time upon primary transmission (1,044 days post-infection, d.p.i.) for the preparation of the inoculum. This led to the appearance of overt clinical signs with severe excitability and ataxia. Voles showed 483±85 d.p.i. survival time and 100% transmission rate based on both spongiform change and PrP^Sc^ accumulation; this latter retained the typical signature of BSE (Fig. 3, lane 1).

**Figure 4:**
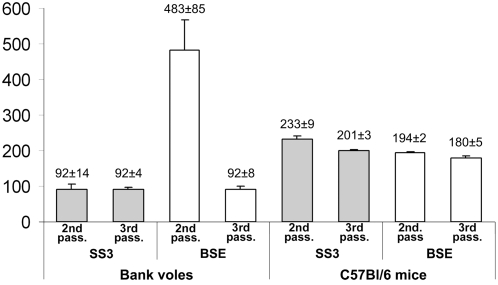
Second and third passages of natural scrapie (SS3) and BSE to bank voles and C57Bl/6 mice. Mean survival times±SD are reported at the top of each bar.

The third passages of SS3 and BSE were carried out in order to investigate their adaptation to bank voles and C57Bl/6 mice (Fig. 4). The survival time was unchanged after the second passage of SS3 in both species. On the contrary, it further shortened in bank voles that were challenged with BSE but not in C57Bl/6 mice. This is likely the consequence of a low level of replication upon primary transmission. This resulted in subclinical infection and possible low infectious titre of the inoculum used for the second passage.

Both SS3 and BSE adapted to voles as very rapid strains, while longer survival times were observed after their adaptation to C57Bl/6 mice (Fig. 4).

### Transmission of 139A to mice and voles shows group-specific patterns

The primary transmission results of natural scrapie and BSE suggested a differential susceptibility of the two vole species on one side, and of C57Bl/6 and wood mice on the other. Bank voles, field voles and wood mice were inoculated with the 139A strain and the transmission characteristics compared to those observed in C57Bl/6 mice in order to investigate if such a pattern of susceptibility was maintained even after the inoculation of a well characterized mouse-adapted scrapie strain.

139A was transmitted very efficiently (100% transmission rate) to all species. All inoculated animals showed clinical signs and revealed spongiform degeneration and PrP^Sc^ accumulation in their brain (Table 2).

**Table 2:** Primary transmission and second passage of 139A to bank voles, field voles, and wood mice.

Recipient species	Primary transmission					Second passage	
	Clin. signs (+)	Pathol. (+)	PrP^Sc^ (+)	Survival time (days±SD)	Transm. rate	PrP^Sc^ (+)/inoculated	Survival time (days±SD)
Bank voles	19/19	19/19	19/19	134±14	100	7/7	75±11
Field voles	8/8	8/8	8/8	126±10	100	7/7	87±7
Wood mice	11/11	11/11	11/11	191±25	100	9/9	149±23
C57Bl/6 mice						9/9	159±3

The transmission of 139A to C57Bl/6 mice is shown in comparison with the second passage to the other species.

Strikingly, both vole species showed shorter survival times than C57Bl/6 mice which is the species to which that strain is adapted. Wood mice showed the longest survival times among the four species (Table 2). Clinical signs of disease were similar in C57Bl/6 and wood mice and characterized by progressive weight loss, dorsal kyphosis, incoordination of hind limbs and plastic tail. The clinical picture in voles was clearly different from that observed after inoculation of natural scrapie. It included hyperactivity/excitability, followed by 10–15 days of reduced activity and behavioural depression. Motor dysfunctions were much less evident compared to what was observed after inoculation of natural scrapie.

**Figure 5:**
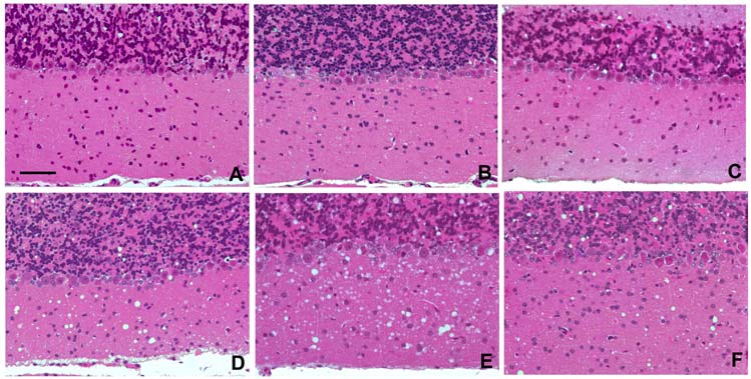
Spongiform change in the cerebellar cortex of rodent species following inoculation of 139A. (A) bank voles, (B) field voles, (C) oldfield mice, (D) wood mice, (E) gerbils and (F) C57Bl/6 mice. Spongiosis is widespread in both molecular and granular layers of wood mice, gerbils and C57Bl/6 mice. In contrast, the cerebellar cortex is quite completely spared in the other three rodent species in which occasional vacuoles were observed only in the granular layer. Bar = 50 µm.

Spongiosis was widespread in the brain of all species with the exception of the cerebellar cortex. Both granular and molecular layers of cerebellar cortex were targeted by moderate/high vacuolar degeneration in C57Bl/6 and wood mice, while spongiosis was only occasional and confined to the granular layer in field and bank voles (Fig. 5).

Interestingly, the molecular analysis of PrP^Sc^ provided further evidence of the differences in the transmission features of prions between voles and mice. As a matter of fact, the typical 139A glycoprofile in mice, monoglycosylated>diglycosylated>unglycosylated, was faithfully maintained in wood mice, while it clearly changed to a diglycosylated>monoglycosylated>unglycosylated pattern in voles (Fig. 1 and 2).

Second passage of 139A was carried out in the three rodent species under investigation. Survival times were very short in both vole species, while in wood mice they were rather long and similar to those observed in C57Bl/6 mice (Table 2). Molecular analysis showed that the PrP^Sc^ glycoprofiles seen in primary transmissions were maintained upon second passages (data not shown).

The adaptation of 139A confirmed the very short survival times of vole-adapted strains, which were already observed with SS3 and BSE. The hypothesis of a high expression level of PrP^C^ which accounted for these findings, was ruled out by Western-blot and Histo-blot analyses, because they did not show any significant differences either in the distribution or in the level of PrP^C^ expression in the brain of bank voles, field voles, wood mice and C57Bl/6 mice (data not shown).

### Back passage of 139A from bank voles to mice demonstrates that the glycosylation pattern of PrP^Sc^ is species-dependent and revertible

139A was fully adapted and stabilized in bank voles with the third passage and subsequently inoculated back into C56Bl/6 mice in order to investigate if the novel PrP^Sc^ glycoprofile observed in voles inoculated with 139A could have been considered as the emergence of a different strain with a new stable molecular signature. The third passage of 139A to bank voles produced the same survival time (76±8 d.p.i.) and PrP^Sc^ characteristics (data not shown) as the second passage. This suggested that the strain had already been adapted to the new host at the second passage.

After inoculation, all C57Bl/6 mice (n = 20) developed the disease showing spongiform change and PrP^Sc^ accumulation in their brain. Survival times were long (463±62 d.p.i.), suggestive of the existence of a transmission barrier also during the transmission from voles to mice, the species to which 139A was originally adapted. Worth mentioning is the fact that the molecular characteristics of PrP^Sc^ reverted to that of the original mouse inoculum (Fig. 3, lane 5).

### PrP sequence analysis suggests a major role of Y154N and S169N substitutions in the transmission of prions to rodent species

**Figure 6:**
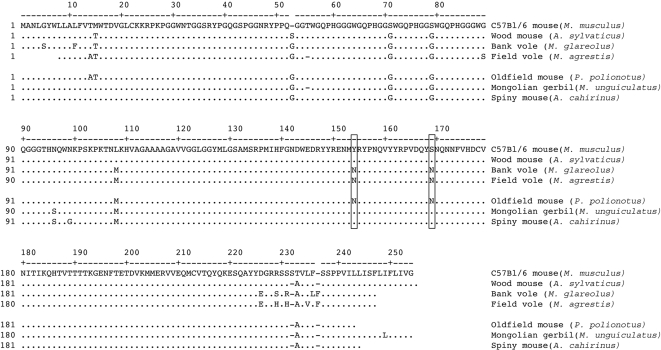
Prion protein amino acid sequences alignment of the rodent species under study. The sequence numbers of C57Bl/6 mouse (*Mus musculus*) amino acids are shown in the upper line and refer to the residue under the first digit. In the other rodent species, residues identical to the mouse are indicated as dots. The amino acid residues that were identified as potentially important for the different susceptibility of vole- and mouse-related species are boxed (154 and 169). Blank spaces at the N or C termini of the sequences represent presently undetermined amino acid residues.

The comparison of PrP sequences of the bank vole, field vole, wood mouse and laboratory mouse displayed a high homology degree, although a number of substitutions were found in the N-terminal cleaved signal peptide and in the C-terminal signal sequence that is also cleaved when the GPI-anchor is added. Sequence comparison showed relevant amino acid substitutions at only five positions (Fig. 6). For the sake of clarity, the numbering system used throughout the text for amino acid residues refers to the mouse PrP sequence.

The first substitution, G89S, was at the N-terminus non-structured tail of PrP and was observed only in the field vole. The second replacement, L108M, was in the N-terminal disordered tail and is known to influence the susceptibility of both voles [8] and mice [10].

**Figure 7:**
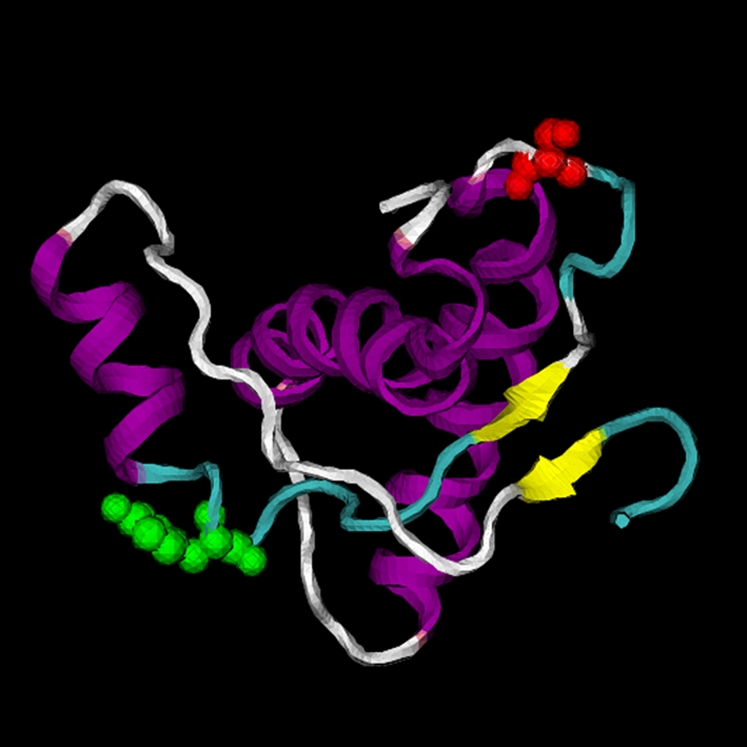
Position of amino acid residues Y154 (green) and S169 (red) in the NMR-structure of the globular domain of mouse PrP^C^. Different secondary structure elements are drawn in different colours (purple: α-helix, yellow: β-sheet, green: loop).

Two substitutions were found in the structured C-terminal domain. A replacement Y154N was found in the loop region between the first α-helix and the second β-strand, while a substitution S to S169N was in the loop between the second β-strand and the second α–elix (Fig. 7). They both distinguished the sequences of laboratory and wood mice from those of voles. Finally, the substitution D226E was in the C-terminal region, and also differentiated the PrP sequences of laboratory and wood mice from those of the two vole species.

We especially focused our attention on the two variations observed in the structured C-terminal domain of PrP, which are located into regions that supposedly contribute to the species barrier because they apparently function as selective protein-protein interaction sites or are involved in the specificity of intermolecular interactions [11].

**Figure 8:**
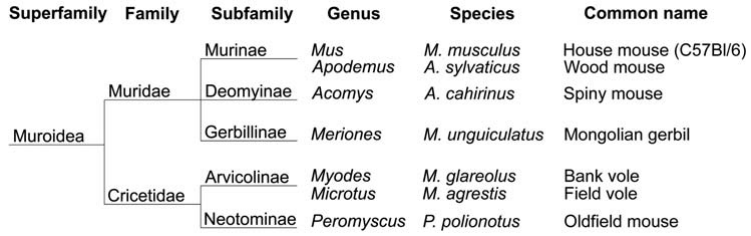
Taxonomic tree of the Muroidea superfamily comprising the rodent species used in the study. All species with Y154–S169 residues (gerbil, C57Bl/6, wood and spiny mouse) fall in the family of Muridae, while species with 154N–169N (oldfiled mouse, bank and field vole) fall in the family of Cricetidae.

In order to test the hypothesis of the role of Y154N and S169N substitutions in influencing the transmission and phenotype characteristics of prions to rodents, we analyzed the PrP sequence of other rodents frequently bred under laboratory conditions and hence selected for transmission studies three additional species: the oldfield mouse, the Mongolian gerbil and the spiny mouse. They were chosen because oldfield mice showed Y154N and S169N substitutions, whereas gerbil and spiny mice did not show them (Fig. 6). Furthermore they have different levels of phylogenetic relationship with the previously inoculated rodent species (Fig. 8) [12].

Groups of oldfield mice, spiny mice and gerbils were challenged with the same inocula of 139A and natural scrapie used in previous transmissions. Following inoculation of both scrapie sources, oldfield mice developed the disease with short survival times, comparable to those of voles, while gerbils showed a very inefficient transmission of natural scrapie and long survival times after inoculation of 139A (Table 3). Overall results confirmed that Y154N and S169N were the only variations that correlated with the different transmission patterns observed. Besides the vole species, residue 108M also occurred in gerbils and spiny mice, while D226E was found in the two voles, but not in oldfield mice which showed a similar susceptibility to voles. N99G was exclusive to spiny mice and might explain the apparent resistance of this species to both 139A and natural scrapie. In fact, this amino acid substitution has been reported to have an inhibitory effect on PrP^Sc^ formation in rabbits, a species thought to be resistant to TSEs [13].

**Table 3:** Primary transmission of 139A and natural scrapie to oldfield mice, spiny mice and gerbils.

Recipient species	Inoculum	Clin. signs (+)	Pathol. (+)	PrP^Sc^ (+)	Surv. time (days±SD)	Transm. rate
Oldfield mice	139A	10/10	8/8	9/9	151±8	100
	SS3	12/12	12/12	11/11	206±23	100
Spiny mice	139A*	0/10	0/1°	0/1°	>1800	0
	SS3*	0/7	0/7	0/7	>1300	0
Mongolian gerbils	139A	5/5	5/5	5/5	238±12	100
	SS3*	1/5	1/1	1/1	1032; >1300	20

*: Experiment in progress. In these cases, the time elapsed from inoculation to writing is reported as survival time.

**°:** No pathological changes or PrP^Sc^ accumulation were detected in the brain of one spiny mouse which was sacrificed 1021 days after inoculation for intercurrent disease (tumour).

In accordance with the overall data, phenotypic analysis of this second set of transmissions by brain histopathology and molecular analysis of PrP^Sc^ revealed characteristics which paralleled those observed in voles, wood mice and laboratory mice. A severe vacuolar degeneration of molecular and granular layers of the cerebellum was evident in gerbils, while the molecular layer was completely spared in oldfield mice (Fig. 5). Furthermore, the glycoprofile of 139A changed in oldfield mice similarly to that previously observed in voles, with the di-glycosylated band appearing the most prominent while it retained the mice-like pattern in gerbils (Fig. 3).

Finally, since we observed that scrapie and BSE adapted to bank voles as much faster strains than in mice, we checked if this also applied to oldfield mice by setting up the second passage of SS3 in that species. The survival time of SS3 in oldfield mice was indeed short (103±11 d.p.i.) and comparable to that observed in bank voles.

## Discussion

We showed that the rodents under investigation can be subdivided into three groups. The first included voles and the oldfield mouse and was characterized by: i) high susceptibility to scrapie, ii) low susceptibility to BSE, iii) extremely short incubation times with adapted strains and iv) change in the 139A glycoprofile. The second group comprised C57Bl/6 mice, wood mice and gerbils and displayed: i) low susceptibility to scrapie, ii) relatively high susceptibility to BSE, iii) longer incubation times with adapted strains and iv) no change in the 139A glycoprofile. The third group included only spiny mice which showed a distinctive resistance to prions.

These findings were consistent with the inefficient transmission of natural scrapie to wild type mice reported by several authors [14],[15], but they also confirmed old observations by Chandler and Turfrey [16], who reported that 50% of field voles inoculated with a rat- or a mouse-passaged scrapie isolate died after 2.5 months, well before the other rodent species which were also challenged.

PrP sequence comparison indicated that Y154N and S169N correlated with the different transmission patterns observed. Overall, species with Y154–S169 were resistant to scrapie, permissive to BSE and reproduced a mouse-like phenotype when infected with 139A, while species with 154N–169N displayed rather opposite features.

The inverted susceptibility of rodents to scrapie and BSE underlined the role of strains in the transmission barrier: amino acid exchanges could either enhance or reduce the efficiency of transmission, depending on the prion strain. In particular, we showed that Y154N–S169N exchanges, which appeared to confer in vole-related species a high susceptibility to scrapie, had the opposite effect with BSE. This is concordant with *in vitro* studies showing that the alteration of the conversion efficiency induced by Y154N–S169N mutations in the vole PrP is strain-dependent, leading to differential effects with vole-adapted BSE and scrapie [9].

The change in the 139A glycoprofile further corroborated the distinction between vole- and mouse-related species. It is known that the PrP^Sc^ glycoform pattern is not necessarily preserved upon interspecies transmission [7],[17]. This may suggest that the glycoprofile is a phenotypic characteristic which is not intrinsic to strains, but it might also reveal a more general phenomenon of strain components selection during interspecies transmission [18]. The recovery of the original 139A glycotype after back passage from voles to mice demonstrated that the change did not imply a permanent mutation, thus suggesting a possible direct effect of Y154N and S169N variations in the PrP sequence of the recipient species on this strain-related characteristic. This confirms previous observations that the glycosilation pattern of PrP^Sc^ can be also influenced by the host [7],[17].

Piening et al [9] analyzed the role of PrP sequence by an *in vitro* conversion assay in the aim to investigate the basis of the higher susceptibility of bank voles to natural scrapie in comparison to mice. In agreement with our *in vivo* results, *in vitro* studies identified the Y154N and S169N substitutions as being responsible for the different conversion efficiency obtained with mouse and vole PrP^C^. Notably, ovine and murine PrP have the same amino acids at positions 154 and 169, while bovine PrP differs only at codon 154, having H instead of Y. In agreement with such differences, *in vitro* assays showed that the vole PrP^C^ was less efficiently converted than that of mouse by both scrapie and BSE [9]. However, by introducing the murine double mutation 154N–169N into the bank vole sequence, the conversion efficiency was enhanced up to a level comparable to the efficiency achieved with mouse PrP^C^, irrespective of remaining mismatches at residues 108 and 226. These findings suggested that the similarity at positions 154 and 169 represented a major determinant of species barrier between the above species. Nevertheless, the different conversion efficiency of mouse and vole PrP^C^ by sheep scrapie did not correlate with the *in vivo* susceptibility of the two species. Assuming that the conversion of PrP^C^ is caused by a direct interaction with PrP^Sc^ [19], *in vitro* studies implied that the recognition and conversion of mouse PrP^C^ by sheep PrP^Sc^ were more efficient than those of vole PrP^C^. However, our *in vivo* results suggested that other factors subsequent to such interaction might have influenced the pathogenesis, leading voles to develop the disease more easily than mice.

On this basis, it is tempting to speculate that voles allow a particularly efficient adaptation and/or rapid replication of prions, as suggested also by the unusually short incubation times of adapted strains. This latter was a striking feature of bank vole, given that almost all vole-adapted prions showed survival times ranging from ∼35 to 130 d.p.i., irrespective of whether they derived from humans, cattle, sheep, deer, mice, or hamsters ([7],[9]; Agrimi, unpublished observations). Herein, we showed that this feature also applies to field voles and oldfield mice. In these rodents the second passage of SS3 produced survival times comparable to bank voles. Furthermore 139A induced disease with survival times shorter than in the donor species, C57Bl/6 mice, even upon primary transmission.

The Syrian hamster model has represented a major advance in prion research owing to the extremely short incubation period of the hamster-adapted strain 263K [20]. Interestingly, both hamsters and voles are 154N–169N. However, the comparison of their susceptibility leads to contrasting observations. It is known that hamsters resist BSE challenging [21], similarly to voles. Furthermore we found that the glycoprofile of the 139H hamster strain, which derived from mouse 139A [22], is shifted toward diglycosylated PrP, similarly to voles (data not shown). On the other hand, our attempt to transmit SS3 to hamsters was unsuccessful [9]. The presence of amino acids that are unique to hamster species (V111M, I138M, V202I, M204I, V214T) offers a potential explanation for these discrepancies. Indeed, at least in the case of 138M, Priola and Chesebro [23] demonstrated in a cell system that this single hamster-specific residue could influence the transmission barrier between mouse and hamster by blocking the conversion of PrP.

The molecular basis of interspecies transmission and adaptation of prions are unknown. Nevertheless, evidence suggests that the PrP sequence of the recipient species acts by dictating the range of possible PrP conformations and hence conditioning the susceptibility to different prion strains [3]. According to this model, the vole sequence would be particularly prone to adopting a wide range of conformations. This would explain the high susceptibility of voles to a variety of TSEs upon primary transmission, although with important exceptions such as BSE. In agreement with the low efficiency of transmission of BSE, also vCJD, which derives in humans from infection by the BSE agent, showed in bank voles very low transmission rate and extremely long survival time (Agrimi, unpublished observations). This supports the idea that the BSE agent transmits poorly to species carrying the Y154N–S169N substitutions, irrespective of the PrP sequence of the donor species.

Positions 154 and 169 are quite variable among mammalian PrPs. Human and bovine sequences are 154H–169S, sheep and goat 154Y–169S, elk and deer 154Y–169N. Considering the strain-related effect of variations at these positions, it could be speculated that such differences may account for the apparent limitation of prion interspecies transmission observed among humans, cervids and small ruminants. Actually, the only TSE proven to have crossed a species barrier naturally is BSE, which transmitted from cattle to humans; two species that share the same amino acids at positions 154 and 169.

In the three-dimensional representation of mouse PrP^C^, residues 154Y and 169S, corresponding to 154N and 169N of the vole prion protein, are exposed on the protein's surface (Fig. 7) and are therefore accessible for potential interactions with PrP^Sc^. Interestingly, it has been shown that also in a model of PrP^Sc^ based on electron micrographs of two-dimensional crystals [24], 154Y–169S residues are located on accessible surfaces of the β-helical core structure potentially important for PrP^Sc^-fibril formation [9]. Interestingly, position 169 lies in the loop connecting the second β sheet and the second α helix (β2-α2) (Fig. 7), a region which is critical in conditioning the PrP^C^ three-dimensional structure [25], the formation of fibrils [26], the susceptibility of sheep to scrapie [27]–[29], the replication of prions [30] and the transmission barrier ([11],[19], present paper). Furthermore, 169N has recently been identified as controlling the conformational plasticity of the β2-α2 loop [31]. As a whole, these data highlight the relevance of these positions when modelling the interspecies barrier. For instance, this could be significant when estimating the risk of prions for humans in primate models, which show a high variability at position 154 and in the β2-α2 loop, including position 169.

The distinction between vole- and mouse-related species inferred by transmission studies is paralleled by the taxonomy, which classifies voles and oldfield mice in the family of Cricetidae, while the remaining species in that of Muridae [12]. This suggests the need to consider the possible existence of host factors in addition to PrP which differently modulate the transmission barrier in the Cricetidae and Muridae families.

Finally, our study showed that the range of rodent models with improved susceptibility to TSEs is wider than it has appeared in studies up to date. Moreover, the high susceptibility of voles and oldfield mice to TSEs gave rise to questions about the possible role of wild rodents in the natural spread of animal TSEs suggesting an intriguing field for epidemiological investigations.

## Materials and Methods

### Animals

Bank voles (*Myodes glareolus*, formely *Clethrionomys glareolus*), field voles (*Microtus agrestis*), wood mice (*Apodemus sylvaticus*), oldfield mice (*Peromyscus polionotus*) and spiny mice (*Acomys cahirinus*) were obtained from breeding colonies at the Istituto Superiore di Sanità, Rome, Italy. Mongolian gerbils (*Meriones unguiculatus*) and house mice (C57Bl/6) (*Mus musculus*) were purchased from Charles River (Como, Italy). The research protocol was approved by the Service for Biotechnology and Animal Welfare of the Istituto Superiore di Sanità and authorized by the Italian Ministry of Health, according to Legislative Decree 116/92, which implemented the European Directive 86/609/EEC on laboratory animal protection in Italy. Animal welfare was routinely checked by veterinarians from the Service for Biotechnology and Animal Welfare.

Subjects were individually identified by passive integrated transponders, inoculated when weanlings (40–60 days) and kept in groups of two-four individuals per cage.

### Inocula

Scrapie-infected brain tissue (SS3) was obtained from the thalamus of a naturally-affected sheep of Sarda breed from Tuscany, which carried the AA_136_RR_154_QQ_171_ PrP genotype. The mouse-adapted scrapie strain 139A was kindly provided by Prof. M. Pocchiari (Istituto Superiore di Sanità). The BSE inoculum was prepared from the *medulla oblongata* of clinically-affected cattle diagnosed in Italy in 1994. All inocula consisted of 10% (w/v) brain homogenate in sterile saline.

### Inoculations and clinical follow-up

Animals were anaesthetized with ketamine and inoculated intracerebrally (i.c.) into the left hemisphere with 20 µl brain homogenate. Beginning one month after inoculation, animals were examined twice per week until the appearance of clinical symptoms, and then examined daily. We measured the survival time instead of the incubation time because of the differences among species in the clinical phenotype of the disease. Diseased animals were sacrificed with carbon dioxide at the terminal stage of disease but before neurological impairment was such as to compromise welfare and, especially, adequate drinking and feeding. Survival time was calculated as the interval between inoculation and sacrifice or death.

### Histopathology, immunohistochemistry and Western-blot analysis

After collection at sacrifice, each brain was cut parasagitally into two parts. The smaller one was stored at −80°C for biochemical studies. The other part was fixed in formalin for histology and immunohistochemistry analysis as described previously [7]. Total PrP as well as PK-resistant PrP were examined by Western blotting in SDS-PAGE gels, as previously described [7].

### PrP sequence determination

Genomic DNA was extracted from frozen brain samples using standard procedures. The coding region of the PrP gene from each species was amplified from 100 ng of genomic DNA using the polymerase chain reaction (PCR). PCR reactions were performed with either MoPrP5 (TGGGCACTGATACCTTGTTCCTC) and MoPrP3 (CCCAGCCTAGACCACGAGAATG) primers (wood mouse) or PrP5uni (TYAGYCATCATGGCRAACCTTRGC) and PrP3uni (TCATCCCACBATCAGGAAGATGAG) (bank and field voles). The latter primers were moderately degenerated on the basis of known rodent PrP sequences and located within the coding region of the PrP gene. The purified PCR products were re-amplified in a ‘nested’ PCR to attach sequences corresponding to standard sequencing primers. The re-amplified products were cycle sequenced using Thermo sequenase (Amersham Pharmacia, Freiburg, Germany) and 5′-IRD-800 labelled primers according to the manufacturer's recommendations. Sequences were determined with the help of an automated system (Model 4000L, LI-COR, Lincoln, NB).

The spiny mouse, the oldfield mouse and the bank vole PrP coding regions were successfully amplified with primer C1-for (TGTAAAACGGCCAGTCCTCATTTTGCAGATCAG) and C1-rev (CAGGAAACAGCTATGACCGGTCCTCCCAGTCATTGCC) or with C2-for (TGTAAAACGACGGCCAGTGGCACCCACAATCAGTGG) C2-rev (CAGGAAACAGCTATGACCCACGATCAGGAAGATGAG). Details on the primers and PCR conditions are available from the authors upon request. PCR products were purified and sequenced with the Big Dye primer cycle sequencing kit (Applied Biosystems, CA, USA). Sequences were determined with an ABI Prism 310 apparatus (Applied Biosystems).

The PrP sequence of gerbils was obtained from GenBank (AF117314).

### Accession numbers

The GenBank (http://www.ncbi.nlm.nih.gov/Genbank) accession numbers for the prion proteins discussed in this paper are: C57Bl/6 mouse (M18070), wood mouse (AF367623), spiny mouse (EF467171), bank vole (AF367624), field vole (AF367625), oldfield mouse (EF467170), Mongolian gerbil (AF117314), Syrian hamster (M14054). The Protein Database (PDB) accession number of mouse PrP^C^ in Figure 7 is 1AG2.
